# The Functional Crosstalk between HER2 Tyrosine Kinase and TGF-*β* Signaling in Breast Cancer Malignancy

**DOI:** 10.1155/2011/804236

**Published:** 2011-02-24

**Authors:** Shizhen Emily Wang

**Affiliations:** Division of Tumor Cell Biology, Beckman Research Institute of City of Hope, KCRB-2007, 1500 East Duarte Road, Duarte, CA 91010, USA

## Abstract

Accumulating evidence indicates a functional crosstalk between the HER2 (ErbB2) tyrosine kinase and the TGF-*β* signaling mediated by its serine/threonine kinase receptors. In HER2-overexpressing breast cancer, this crosstalk results in increased cancer cell proliferation, survival and invasion, accelerated cancer progression and metastasis in animal models, and resistance to chemotherapy and HER2-targeted therapy. The transformed cellular context with constitutively active HER2 signaling, as a consequence of HER2 gene amplification or overexpression, converts TGF-*β* from a tumor suppressor to a malignancy-promoting factor. TGF-*β*, in turn, potentiates oncogenic HER2 signaling by inducing shedding of the ErbB ligands and clustering of HER2 with integrins. In addition, TGF-*β* is associated with resistance to trastuzumab, an anti-HER2 therapeutic antibody. Recent mechanistic studies indicate that TGF-*β* and HER2 cooperate through both Smad-dependent and independent mechanisms. Blockade of HER2:TGF-*β* crosstalk may significantly enhance the efficiency of conventional therapies in breast cancer patients with HER2 overexpression.

## 1. HER2 Is a Proto-Oncogene and a Therapeutic Target in Breast Cancer

HER2 (ErbB2/Neu) is a member of the ErbB family of transmembrane receptor tyrosine kinases (RTKs), which also includes the epidermal growth factor receptor (EGFR, ErbB1), HER3 (ErbB3), and HER4 (ErbB4). Ligand binding to the ectodomains of EGFR, ErbB3, and ErbB4 results in the formation of catalytically active homo- and heterodimers to which HER2 is recruited as a preferred partner [1]. Although HER2 cannot bind any ErbB ligand directly, its catalytic activity can potently amplify signaling by ErbB-containing heterodimers via increasing ligand binding affinity and/or receptor recycling and stability [2–5]. Activation of the ErbB network leads to receptor autophosphorylation of C-terminal tyrosines and recruitment to these sites of cytoplasmic signal transducers that regulate cellular processes such as proliferation, differentiation, motility, adhesion, protection from apoptosis, and transformation. Cytoplasmic signal transducers activated by this network include PLC-*γ*1, Ras-Raf-MEK-MAPKs, PI3K-Akt-ribosomal S6 kinase, Src, the stress-activated protein kinases (SAPKs), PAK-JNKK-JNK, and the signal transducers and activators of transcription (STAT) [1]. Several RTKs, including the ErbB family members, fibroblast growth factor receptors, insulin receptor, and vascular endothelium growth factor receptor Flk1/KDR, are known to migrate to the nucleus and act as transcription factors for certain target genes [6]. Nuclear HER2 has been found to associate with multiple genomic targets *in vivo*, including the cyclooxygenase enzyme COX-2 gene promoter, and stimulate gene transcription [7].

*HER2* gene amplification is reported in *∼*20% of metastatic breast cancers, where it is associated with poor patient outcome [8]. Studies of HER2-overexpressing breast cancer cell lines and human tumors have shown constitutive HER2 phosphorylation [9, 10]. Overexpression of HER2 is associated with mammary epithelial cell transformation [11, 12] and shorter survival in breast cancer patients [8, 13]. Trastuzumab (Herceptin), a humanized IgG_1_ that binds to HER2 ectodomain, is an approved therapy for treating HER2-overexpressing breast cancers [14, 15]. Trastuzumab has been shown to induce tumor regressions in 12%~35% of heavily pretreated metastatic breast cancers with HER2 overexpression [16–18]. Meanwhile, most metastatic breast tumors with HER2 gene amplification and/or very high levels of HER2 protein do not respond to trastuzumab, and the majority of those that initially respond relapse later, suggesting de novo and acquired mechanisms of therapeutic resistance.

The mechanisms of resistance to trastuzumab are not fully understood. However, recent reports suggest that overexpression of the IGF-I receptor [19] or activated EGFR [20] as well as aberrant PI3K/Akt signaling [21] or PTEN deficiency [22] may all result in acquired resistance to trastuzumab. Lately, intragenic somatic mutations in the HER2 gene were reported in <10% of non-small-cell lung cancers (NSCLCs) [23, 24]. These involve in-frame duplications/insertions in a small stretch within exon 20 that correspond to the identical nine-codon region in exon 20 of the EGFR gene, where duplications/insertions have also been reported. Because of the location of these insertions at the C-terminal end of the C-helix in the tyrosine kinase domain, it has been postulated that they result in a conformational change and shift in the helical axis, thus narrowing the ATP-binding cleft and increasing kinase activity over that in wild-type receptors [23]. HER2 kinase domain mutations within exons 18–22 are identified in 5% of gastric carcinomas, 3% of colorectal carcinomas, and <5% of breast carcinomas from Asian patients [25]. Mechanistic studies indicate that the mutant HER2 induces constitutive transphosphorylation of EGFR and activation of the downstream signal transducers in a ligand-independent manner, resulting in increased tumorigenicity and decreased sensitivity to trastuzumab and EGFR inhibitors in cells carrying these mutations [26].

## 2. HER2 Converts TGF-*β* from a Tumor Suppressor to a Tumor Promoter

The TGF-*β* ligands are a family of multitasking cytokines that play important roles in cell proliferation, lineage determination, extracellular matrix production, cell motility, apoptosis, and modulation of immune function [31]. These ligands bind to a heteromeric complex of transmembrane serine/threonine kinases, the type I and type II receptors (T*β*RI and T*β*RII) [31]. Upon ligand binding to T*β*RII, T*β*RI is recruited to the ligand-receptor complex. This allows for the constitutively active T*β*RII kinase to transphosphorylate and activate the T*β*RI kinase which subsequently phosphorylates the transcription factors Smad2 and Smad3 [32]. Smad2/3 then associate with a common mediator Smad, Smad4, and translocate to the nucleus where as a heteromeric complex, they regulate gene transcription [33]. In addition to Smads, other signaling pathways have been implicated in TGF-*β* actions in the recent studies. These include the extracellular signal-regulated kinase (ERK, MAPK), c-Jun NH_2_-terminal kinase (JNK), p38MAPK, phosphatidylinositol-3 kinase (PI3K), and Rho GTPases (reviewed in [34, 35]). The critical role of these non-Smad pathways on mediating the cellular effects of TGF-*β* remains to be fully characterized.

TGF-*β* was originally reported to induce anchorage-independent growth of mouse fibroblasts [36]. Subsequent studies indicated that TGF-*β* is a potent inhibitor of cell proliferation and therefore, a tumor suppressor [37, 38]. Consistent with its tumor suppressor role, many cancers lose or attenuate TGF-*β*-mediated antimitogenic action by mutational inactivation of TGF-*β* receptors or their signal transducer Smads [39–44]. Studies using transgenic mice with conditional knockout of T*β*RII indicate that loss of T*β*RII in the context of polyomavirus middle T antigen (PyVmT) expression results in a shortened median tumor latency and an increased formation of pulmonary metastases. On the other hand, increasing evidence shows that excess production and/or activation of TGF-*β* in tumors can accelerate cancer progression by a combination of autocrine and paracrine mechanisms, resulting in enhancement of tumor cell motility and survival, increase in tumor angiogenesis and production of extracellular matrix and peritumoral proteases, and the inhibition of immune surveillance mechanisms in the cancer host (reviewed in [34, 35, 45]).

TGF-*β* has been shown to synergize with transforming oncogenes in cancer progression. For example, overexpression of active TGF-*β*1 or active mutant of T*β*RI (Alk5) in the mammary gland of bigenic mice also expressing mouse mammary tumor virus (MMTV)/Neu (ErbB2) accelerates metastases from Neu-induced mammary cancers [46–48]. In transgenic mice bearing PyVmT-expressing mammary tumors, inhibition of TGF-*β* with the soluble fusion protein T*β*RII:Fc results in increased apoptosis of tumor cells and a reduction in both circulating tumor cells and lung metastases [49]. In the same transgenic model, conditional induction of active TGF-*β*1 in mice bearing established mammary cancers increases lung metastases by >10 folds without a detectable effect on mammary tumor proliferation or size [50]. Mice expressing soluble T*β*RII under the regulation of the MMTV/LTR promoter exhibit high levels of the TGF-*β* antagonist in the circulation which suppress metastases from Neu-induced mammary tumors as well as metastases resulting from injected B16 melanoma cells [51].

In breast cancer models, a functional synergy between TGF-*β* and HER2 has been characterized. Exogenous as well as transduced TGF-*β* confer motility and invasiveness to MCF10A nontransformed human mammary epithelial cells (HMECs) stably expressing transfected HER2 [52, 53]. Indeed, a genetic modifier screen in these cells identified TGF-*β*1 and TGF-*β*3 as molecules that cooperate with HER2 in inducing cell motility and invasion [37, 52]. Taken together, these data suggest that oncogenic signals, such as overexpression of HER2, are permissive for TGF-*β*-induced signals associated with tumor cell motility and, potentially, metastatic progression. Inhibition of HER2 with trastuzumab blocks the promigratory effect of TGF-*β* on HER2-overexpressing HMEC [53], suggesting that oncogene function is required for the transforming effect of TGF-*β*.

## 3. The Crosstalk between HER2 and TGF-*β* Occurs at Various Levels

Our recent studies demonstrate that TGF-*β* and HER2 cooperate at various levels, including (1) transcriptional regulation of the Smad target genes and pathways; (2) activation of the PI3K/Akt pathway in a Smad-independent manner; (3) modification of the tumor microenvironment by inducing the secretion of TGF-*β*, ErbB ligands, and angiogenic mediators.

We have utilized a cell culture model overexpressing HER2 (MCF10A/HER2) or empty vector (MCF10A/vec) to investigate synergy of HER2 overexpression and TGF-*β* signaling. A chromatin immunoprecipitation-(ChIP-) based screen was carried out to identify chromatin Smad targets (ChSTs) in TGF-*β*-treated MCF10A/HER2 cells [54]. The regulatory regions of several potential TGF-*β* target genes are identified from the ChST DNA pool established in this study. These genes include the receptor-type phosphatase *κ* (PTPRK), serine/threonine kinase 24 (STK24), integrin *α*9 (ITGA9), and vimentin-similar genes. Interestingly, TGF-*β* induces binding of Smads to some of these gene promoters only in MCF10A/HER2 but not in MCF10A/vec cells [54]. This suggests that cofactors regulated by HER2 signaling modulate Smad-mediated transcription and, thereby, the biological functions of TGF-*β* in HER2-overexpressing cells. Further investigation on PTPRK, a Smad target gene indentified in this study, indicates that while TGF-*β* upregulates PTPRK expression in both tumor and nontumor mammary cells, HER2 overexpression downregulates PTPRK. RNA interference of PTPRK accelerates cell cycle progression, enhances response to EGF, and abrogates TGF-*β*-mediated antimitogenesis [54], suggesting a tumor-suppressive role of PTPRK. Therefore, by suppressing PTPRK expression, HER2 abrogates the ability of TGF-*β* to induce antimitotic factors.

Another example of altered regulation of Smad target genes is the mutS homolog 2 (MSH2), a tumor suppressor and central component of the DNA mismatch repair (MMR) system. TGF-*β* upregulates MSH2 expression in non-tumor cells through promoter activation mediated by Smads and p53. However, overexpression of HER2 impairs p53 function and increases the level of miR-21, a microRNA that targets and downregulates MSH2 transcripts [55]. As a result, in HER2-transformed cells, TGF-*β* fails to activate MSH2 promoter but decreases MSH2 expression by further stimulating miR-21 [55]. This downregulation of MSH2 by TGF-*β* also contributes to resistance to DNA-damaging chemotherapy agents in cancer cells, as MSH2 is required for the recognition of drug-induced DNA damages, which triggers apoptosis [55].

**Figure 1:**
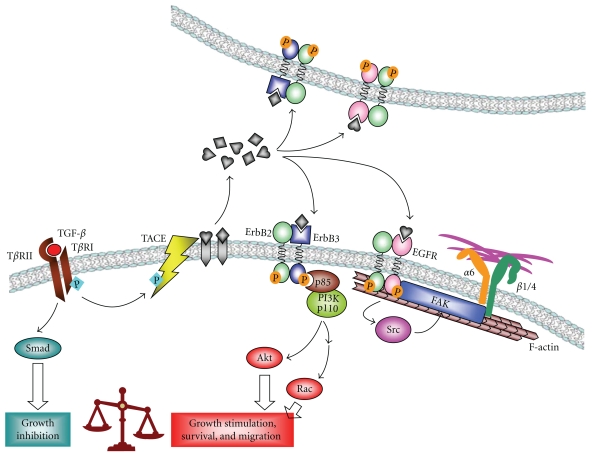
Tumor-promoting function of TGF-*β* in HER2-overexpressing cancer cells is mediated by TGF-*β*-driven autocrine and paracrine ErbB ligands (figure modified from [27]).

In another study, we show that addition of exogenous TGF-*β* or expression of constitutively active T*β*RI (Alk5^T204D^) induces motility of MCF10A/HER2 cells but not MCF10A/vec cells [53]. This is mediated by PI3K activation and involves HER2 translocation to cell membrane protrusions, where it colocalizes with Vav2, Rac1, Pak1, and actin skeleton, resulting in prolonged Rac1 activation and enhanced cell survival and invasiveness [56]. By anchoring HER2 to actin skeleton, TGF-*β* also induces clustering of HER2 and integrin *α*6, *β*1 and *β*4, which is mediated by focal adhesion kinase (FAK) and required for TGF-*β*-induced motility and oncogenic signaling of HER2 in breast cancer cells (Figure 1) [57]. We further investigated the mechanism through which TGF-*β* activates PI3K in HER2-overexpressing cells and found that treatment with TGF-*β* or expression of Alk5^TD^ induces phosphorylation of the TACE/ADAM17 sheddase and its translocation to cell surface, resulting in increased secretion of TGF-*α*, amphiregulin, and heregulin. In turn, these ligands enhance association of PI3K p85 subunit with ErbB3 and activate PI3K/Akt (Figure 1) [27]. In addition, activation of TGF-*β* signaling in HER2-overexpressing breast cancer cells also reduces their sensitivity to trastuzumab, as a result of PI3K activation [27].

While TGF-*β* induces shedding of ErbB ligands into the microenvironment, HER2 signaling also induces the expression and secretion of TGF-*β*1 and TGF-*β*3 through a mechanism involving Rac1 activation and JNK-AP1-dependent transcription [58]. Vascular endothelial growth factor (VEGF), a target of the TGF-*β*-Smad transcriptional regulation, is synergistically induced by HER2 and TGF-*β* [58]. Thus, the crosstalk between HER2 and TGF-*β* not only alters intracellular signaling in cancer cells but also influences other components of the tumor microenvironment through inducing several proinvasive growth factors, which may serve as extracellular targets of novel therapeutic strategies directed at both cancer-driving oncogenes and the modified tumor microenvironment.

## 4. Clinical Relevance of the Crosstalk between HER2 and TGF-*β*

**Figure 2:**
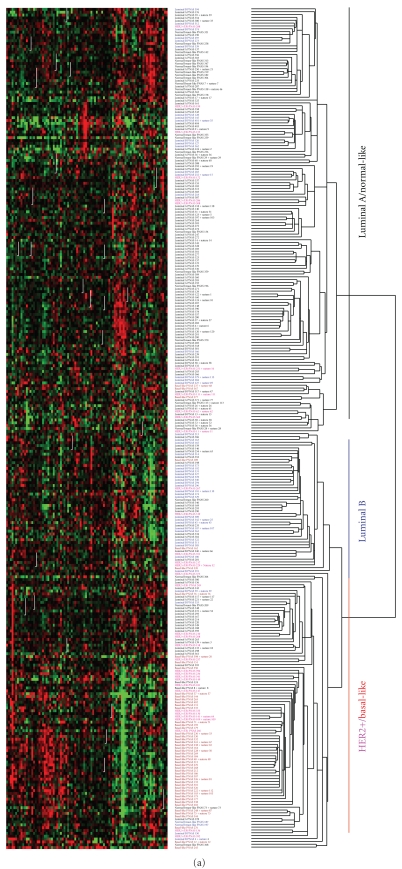

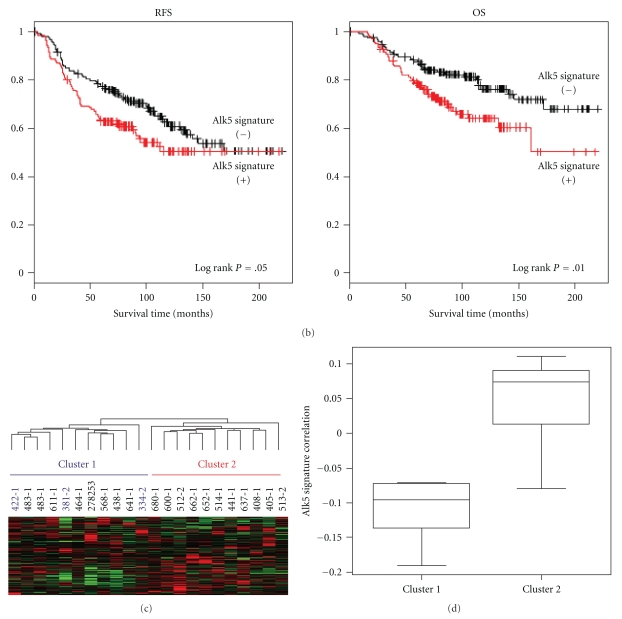
Alk5^TD^ signature is associated with clinical outcome in women with breast cancer (figure adapted from [27]). (a) Hierarchical clustering of 295 breast tumors [28, 29] using 90 overlapping genes with the 271-gene Alk5^TD^ signature. (b) Kaplan Meier plots for recurrence-free survival (RFS) and overall survival (OS) comparing the two groups of tumors with and without a correlation with the Alk5^TD^ signature. (c) Hierarchical clustering of 22 breast tumors from patients who were treated with navelbine and trastuzumab [30] using 190 overlapping genes with the 271-gene Alk5^TD^ signature. Cluster 2 shows a positive correlation with the Alk5^TD^ signature. (d) Box-and-Whisker plot of standard pearson correlation between the Alk5^TD^ signature and clusters determined in (c).

To understand the clinical relevance of the HER2:TGF-*β* crosstalk, we mapped an Alk5^T204D^-induced gene expression signature to a previously published 295-array data set by van de Vijver et al. [29] and Chang et al. [28]. The Alk5^TD^ signature reflects biological and clinical differences in the 295 tumors. The tumors with a positive correlation with the active T*β*RI signature are mostly HER2 positive, Basal-like, and some Luminal B tumors while the tumors with a negative correlation are predominantly Luminal A and normal-like tumors (Figure 2(a)) [27]. Cancers with a positive correlation with the Alk5^TD^ signature show a worse recurrence-free survival (RFS) and overall survival (OS) compared to tumors with a negative correlation (Figure 2(b)). We further explored possible correlation of the Alk5^TD^ signature with resistance to trastuzumab by mapping this gene expression signature to an array data set reported by Harris et al. [30] obtained from 22 patients with HER2-overexpressing breast cancer treated with neoadjuvant trastuzumab and vinorelbine. Hierarchical clustering analysis shows that all 3 patients who achieved pathological complete response do not share similar expression with the TGF-*β* signature (Figures 2(c) and 2(d)) [27], which supports a role of TGF-*β* in inducing clinical resistance to trastuzumab.

As indicated by the studies reviewed herein, the cell readouts of the multifunctional TGF-*β* signaling is context dependent and largely edited by the overexpression of HER2, which is one major dysregulation in breast cancer. In HER2-transformed cells, TGF-*β*, in turn, further stimulates HER2 signaling to promote malignancy and induces resistance to anti-HER2 therapy. Documented evidence suggest that blockage of HER2:TGF-*β* crosstalk may significantly enhance the efficiency of conventional therapies in breast cancer patients with HER2 overexpression.
